# Cocaine-Induced Pneumopericardium: Safe for Discharge? A Case Report and Literature Review

**DOI:** 10.1155/2019/4107815

**Published:** 2019-02-20

**Authors:** Xavier Galloo, Jan Stroobants, David Yeo, Esmael El-Abdellati

**Affiliations:** ^1^Department of Cardiology, ZNA Middelheim, Antwerp, Belgium; ^2^Emergency Department, ZNA Middelheim, Antwerp, Belgium; ^3^Emergency Department, University Hospital Birmingham, Birmingham, UK

## Abstract

A 29-year-old male presented at the Emergency Department (ED) with chest pain and neck tenderness after intranasal cocaine usage. Physical exam of the patient demonstrated moderate subcutaneous emphysema on the right side of his neck. The ECG did not demonstrate any changes associated with cocaine-induced cardiac ischemia, and blood analysis was normal (negative troponins). A chest X-ray revealed subtle evidence of pneumomediastinum. Subsequent thoracic CT confirmed the presence of subcutaneous emphysema with a pneumopericardium and a large pneumomediastinum along with a small pneumothorax. The patient was managed conservatively and kept overnight for observation. He was discharged from the ED the following day with ambulatory follow-up. A repeat thoracic CT performed two weeks later demonstrated that the findings identified in the first CT had resolved. Pneumopericardium, -mediastinum, and -thorax are rare conditions reported after cocaine abuse. A conservative approach with a period of observation in a suitable ambulatory unit is acceptable, as current literature suggests that the condition is usually self-limiting.

## 1. Introduction

The widespread abuse of cocaine has led to an increase in the frequency of Emergency Department (ED) visits worldwide. The respiratory and cardiovascular effects associated with cocaine abuse are well established with increasing numbers reported in the literature. The most common complaint reported by patients is chest pain [1, 2].

Cocaine activates the sympathetic nervous system leading to vasoconstriction, elevated arterial blood pressure, tachycardia, and arrhythmias. The most frequent cardiovascular complications reported are acute myocardial infarction and cardiac tachyarrhythmias. Pneumomediastinum and -thorax after cocaine abuse are reported infrequently, and of note, pneumopericardium is very rare [1, 3].

## 2. Case Report

A 29-year-old male (weight: 58 kg, length: 178 cm, BMI: 18.3 kg/m^2^; BMI normal range: 18.5 kg/m^2^ – 25 kg/m^2^), without significant past medical history, presented at the ED with chest pain and localised neck tenderness. He presented with sudden onset retrosternal chest pain. The pain was described as “mild and continuous” with no radiation or any other associated symptoms (i.e., no dyspnea or cough; no nausea or vomiting). There was no history of preceding trauma, and the patient had no previous medical history of note. Apart from a 5 pack-year history of smoking cigarettes, there were no other cardiovascular risk factors.

The patient did not take regular medication but admitted to marijuana use on a weekly basis as a teenager until the age of 25. He then only used marijuana infrequently (on average once a month). With regard to cocaine, he admitted he had used it twice—the first being a year ago, and the second time a day prior to attending the ED. There was no history of any other recreational drug use.

Following intranasal cocaine inhalation at an evening social gathering, he suffered chest pain and palpitations. In an attempt to ease the chest pain, he smoked a premixed marijuana/tobacco joint (approximately 0.32 grams marijuana [4]). The joint was smoked as per a normal cigarette technique without prolonged inhalation or Valsalva manoeuvres.

The following morning, he continued to experience retrosternal chest pain, which had since increased in intensity. He now noticed the presence of right-sided neck pain but without muscle tenderness, throat pain, or dyspnea. Despite taking 1 gram of paracetamol, the symptoms were no better and he therefore presented to the ED.

His vital signs were within normal parameters (temperature: 36.1°C, heart rate: 65 bpm, blood pressure: 125/75 mmHg, equal bilaterally with no pulsus paradoxus, respiratory rate: 13/min, oxygen saturation 100% on room air with a GCS of 15/15).

On physical examination, respiratory auscultation was normal without additional sounds; cardiac sounds were unremarkable with no murmurs or clear “Hamman's sign” (*a crunching sound synchronous with the heartbeat. It is best heard over the precordium and is suggestive for pneumopericardium or -mediastinum*) [5–7]. A moderate amount of subcutaneous emphysema on the right side of the neck was noted. The rest of the physical exam was unremarkable.

**Figure 1:**
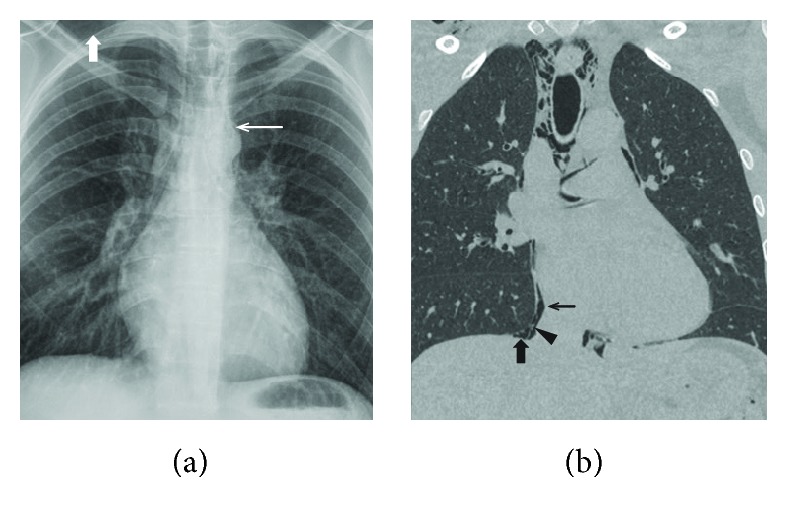
Chest X-ray (a) and chest CT (b). On both pictures, subcutaneous emphysema is apparent at the right side of the neck (thick white arrow) as well as air tracking in the centre of the mediastinum indicative for mediastinal emphysema or pneumomediastinum (thin white arrow). Chest CT reveals a pneumopericardium (thin black arrow) and a small pneumothorax (thick black arrow). The pericardium can be seen between the pneumopericardium and pneumothorax (black triangle).

**Figure 2:**
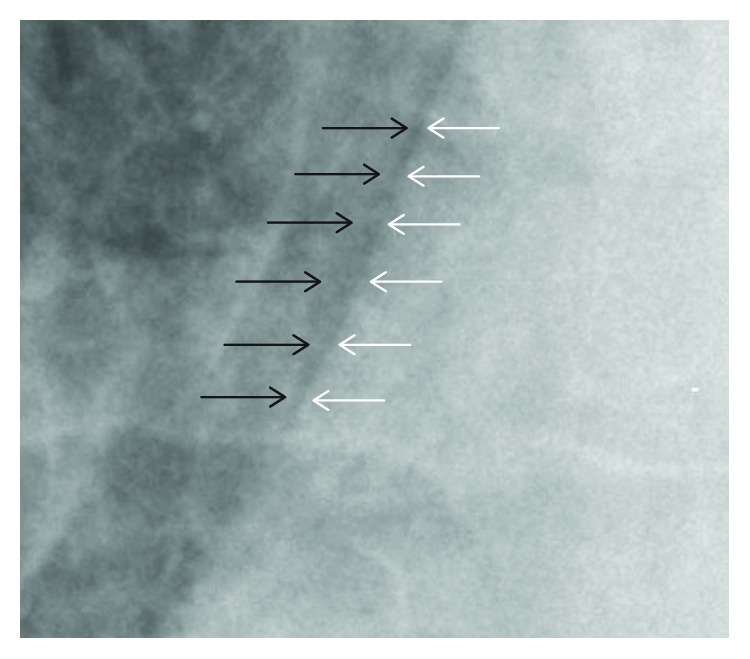
Mach band effect versus pneumomediastinum. Zoom in on the right cardiomediastinal border showing the edge enhancement along the cardiac margin. White arrows depicting the right cardiac border and black arrows depicting the mediastinal border of the right lung.

Blood analysis was normal (normal haematology and biochemical analysis, normal inflammatory markers and troponins <0.012 ng/mL). Arterial blood gas on room air demonstrated a respiratory alkalosis without metabolic compensation (pH 7.50, pCO_2_ 29.3 mmHg, pO_2_ 119.7 mmHg, base excess 0.3 mmol/L, HCO_3_^−^ 22.2 mmol/L, saturation 98.2%, and lactate 1.3 mmol/L). ECG demonstrated normal sinus rhythm without ST-elevation with normal repolarisation. A chest X-ray showed subtle evidence of pneumomediastinum and right-sided mild subcutaneous emphysema in the neck (Figure 1(a), thick arrow) with air tracking in the centre of the mediastinum (Figure 1(a), thin arrow). There is a small denser line along the right cardiomediastinal margin (Figure 2) that is often present in normal X-rays and is attributed to an optical illusion known as the *Mach Band effect* (a visual pattern due to edge enhancement manifesting as a region of lucency adjacent to convex surfaces). Although it can be normal, taken in association with subcutaneous emphysema and air tracking in the centre of a mediastinum, this is suggestive of a pneumomediastinum [8].

**Figure 3:**
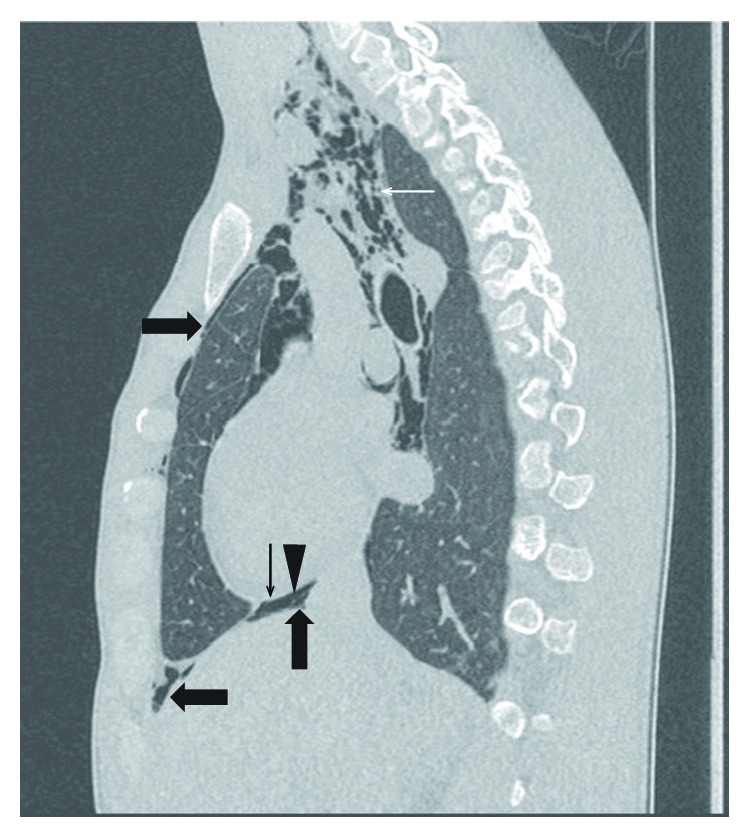
Chest CT showing extensive subcutaneous and mediastinal emphysema compatible with a large pneumomediastinum (white arrow), a pneumopericardium (thin black arrow, with pericardium marked by the black triangle), and a small pneumothorax (thick black arrows).

As subcutaneous emphysema along with the suggestion of pneumomediastinum was identified on the chest X-ray, further workup with thoracic CT-scan was performed. This confirmed the presence of subcutaneous emphysema with a pneumopericardium, a large pneumomediastinum, and a small pneumothorax (Figure 1(b), Figure 3). The patient was managed conservatively and kept overnight for observation (continuous cardiac and pulse oximetry monitoring with two-hourly blood pressure measurement in addition to pain control titrated to a pain Visual Analogue Scale). A combination of paracetamol and tramadol was sufficient to keep the patient pain free. The following day, his clinical parameters remained within normal limits along with the resolution of chest pain and a largely unremarkable physical examination. He was discharged the next day with expectative approach, oral analgesics, and ambulatory follow-up. Repeat CT after two weeks revealed complete resolution of the free intrathoracic air.

## 3. Discussion

Spontaneous pneumopericardium, -mediastinum, and -thorax are rare conditions that have been reported following cocaine abuse, but diagnostic and therapeutic guidelines remain open to debate. The current ESC (European Society of Cardiology) guidelines for pericardial diseases do not describe the management of pneumopericardium in this context [9, 10]. Pneumopericardium is an extremely rare complication of cocaine abuse with only 9 published cases to the best of our knowledge [11–18]. It is unclear what the incidence, or indeed the relationship, between the use of cocaine and spontaneous pneumopericardium is. A study with a systematic toxicology screening in patients presenting with spontaneous pneumopericardium/pneumothorax might shed some light on this.

Pneumopericardium, -mediastinum, and -thorax are defined as the presence of “free” air in the pericardium, the mediastinum, or the pleural cavity, respectively [7, 19]. Where there is no clear aetiology, it is referred to as spontaneous/primary, and secondary when a specific pathologic event (trauma, infection, and iatrogenic) is thought to be causative [6]. Cocaine-related pneumopericardium, -mediastinum, and -thorax are considered as spontaneous or primary as determining the precise aetiology of “free” air is challenging and its mechanism remains unanswered.

It is unclear whether the pathophysiology of pneumopericardium is that of pulmonary barotrauma or microscopic tracheal/oesophageal tear due to the solid contaminants in the crystalline mass inhaled or snorted by the patient [11, 15]. Pulmonary barotrauma, also known as the *Macklin effect*, can be explained by a three-step process: alveolar rupture due to an abrupt increase in intra-alveolar pressure leading to air dissection along bronchovascular sheaths, with eventual spread to the pulmonary interstitium and the mediastinal and pericardial cavity [7, 20–22]. This is thought to be the result of either a sudden increase in intrathoracic pressure due to rapid nasal inhalation, coughing, sneezing, and vomiting or deep, forced, and prolonged inhalation with a Valsalva's manoeuvre [1, 3].

The patient denied any coughing, vomiting, prolonged inhalation, or Valsalva's manoeuvre. Our hypothesis is that the pneumopericardium, -mediastinum, and -thorax in this patient were the result of a sudden rise in intra-alveolar pressure due to cocaine inhalation. As the chest pain preceded the smoking of marijuana, it is believed that marijuana was far less likely to be the cause of the pneumopericardium, -mediastinum, and -thorax.

In the published literature, the majority of the patients presenting with spontaneous pneumopericardium and/or pneumomediastinum are young thin males without previous medical history, much like this patient. The most frequent complaint described is chest pain, followed by neck pain, dyspnea, and cough. Less frequent symptoms are odynophagia, hoarseness, and feeding problems [7, 9, 15, 17]. Emergency physicians should be alert for patients presenting with these symptoms as they have a considerable higher risk of developing pneumopericardium and/or pneumomediastinum.

Spontaneous pneumopericardium and -mediastinum can be diagnosed on plain chest X-ray; although, thoracic CT remains the gold standard for radiological investigations. A CT-scan can also be helpful in excluding secondary/other causes of pneumopericardium. Further invasive diagnostic studies are not normally needed in spontaneous pneumomediastinum and should only be performed in highly suspicious cases of oesophageal or tracheal rupture [9]. There have been no reports of oesophageal rupture (Boerhaave syndrome) associated with cocaine-induced pathology. As our patient did not complain of gastrointestinal symptoms, there was no indication for invasive diagnostics such as oesophagoscopy/gastroscopy or bronchoscopy. Where tamponade due to the pneumopericardium is suspected bedside, echocardiography may be useful in evaluating such patients. As our patient remained stable throughout the entire observation with no evidence of pulsus paradoxus, echocardiography was not required.

Some guidelines for spontaneous pneumothorax and pneumomediastinum recommend a conservative approach with outpatient follow-up in selected patients with minimal or no symptoms. These patients should receive clear advice to return in case of worsening dyspnea, chest pain, or fever [19, 23]. The treatment of spontaneous pneumopericardium is not clearly defined due to the rarity of its presentation. It is generally considered a benign condition, which responds well to conservative treatment, consisting of bed rest, oxygen therapy, and analgesia. All reported cases treated conservatively have resulted in complete resolution over a period of one to two weeks. Oxygen therapy should be considered as its use increases the diffusion pressure of nitrogen in the interstitium, promoting the absorption of free air in the mediastinum and possibly in the pericardium [7, 9]. In the referenced literature, there were no reports of patients developing pericarditis or mediastinitis. Antibiotic treatment should only be considered in patients at risk for infective pericarditis (presenting with fever, blood sample showing elevated inflammatory parameters, or pneumopericarditis secondary to oesophageal rupture) and should not normally be given empirically [9].

## 4. Conclusion

Cocaine-related complaints are increasingly seen in the ED as cocaine abuse increases in the community. The most common complaint of cocaine abuse is chest pain. Although pneumomediastinum and pneumothorax after cocaine abuse has been reported, they are infrequent complications; pneumopericardium is even less common and poorly documented. Whilst the emergency physician is familiar with the most common causes (acute myocardial ischemia or arrhythmia) of chest pain after cocaine abuse, they should be aware of other rarer causes. Patients meeting the criteria of a young thin male with no previous medical history are at increased risk of developing pneumopericardium and/or pneumomediastinum. When the ECG shows no signs of cardiac ischemia or arrhythmia and serial cardiac marker testing are negative in asymptomatic, or mildly symptomatic, patients with spontaneous pneumopericardium, a short observation with monitoring in a Chest Pain Unit is advised to follow-up or promptly exclude and treat life-threatening complications (i.e. tension pneumothorax or tamponade) early, followed by an expectative approach, since most patients will have spontaneous resolution within one or two weeks.

Each case needs to be managed on its own merits based upon the history, physical examination, investigations, clinical course of the patient, and clinical judgment of the treating physician. In some cases, prolonged observation may be necessary.
